# Supplementary material for: Association between Self-Reported Prior Night’s Sleep and Single-Task Gait in Healthy, Young Adults: A Study Using Machine Learning
Source: Sensors (Basel). 2022 Sep 29;22(19):7406. doi: 10.3390/s22197406 (PMC9572361; doi:10.3390/s22197406)
Supplement: Supplementary file 1 [file sensors-22-07406-s001.zip › sensors-1903616-supplementary.pdf]

Supplementary Table S1: Complete set of gait variables

Variable (units)
Lower Limb Variables
Mean cadence (step/min)
Cadence variance (%)
Mean toe out angle (deg)
Variance mean toe out angle (%)
Mean foot strike angle (deg)
Variance foot strike angle (%)
Mean circumduction (cm)
Variance circumduction (%)
Mean stride length (m)
Variance stride length (%)
Mean step variability
Mean mid-swing elevation (cm)
Variance mid-swing elevation (%)
Mean double leg support (%GCT)
Asymmetries double leg support (%)
Upper Limb Variables
Mean upper arm ROM (deg)
Variance upper arm ROM (%)
Turning Variables
Mean turn angle (degrees)
Mean steps in turn (#)
Mean turn velocity (degrees/s)
Anticipatory Postural Adjustment
Forward APA Peak (m/s2)
Lateral APA Peak (m/s2)
First Step Range of Motion (deg)
APA Duration (s)
**Trunk**
Neck – Mean Sagittal ROM (degrees)
Neck - Mean Coronal ROM (degrees)
Neck - Mean Transverse ROM (degrees)
Back - Mean Sagittal ROM (degrees)
Back - Mean Coronal ROM (degrees)

Back - Mean Transverse ROM (degrees)

Abbreviations: APA = anticipatory postural adjustment; GCT = ground contact time; ROM = range of motion
